# Pathological Histology

**Published:** 1847-07

**Authors:** 


### Art. VII.

*Die Pathologische Gewebelehre von* Dr. Friedrich Günsburg. Erster Band. *Die Krankheitsprodukte nach ihrer Entwickelung, Zusammensetzung, und Lagerung in den Geweben des menschlichen Körpers.* Mit drei Tafeln. *Leipzig,* 1845.

*Pathological Histology.* By Dr. F. Günsburg. First Volume. *On the Development, Connexion, and Deposition of Morbid Products in the Tissues of the Human Body.* With three Plates.—*Leipsic,* 1845. 8vo, pp. 287.

Several of the German journals have, for some years past, afforded evidence of the industry and accuracy of Dr. Günsburg as a microscopic investigator; and we gladly welcome the appearance of the present volume, as affording us the means of laying before the readers of this Journal the results of some of his most important labours.

The ordinary reader will undoubtedly regard the book as dry and uninteresting; it is exclusively devoted to the microscopic examination of morbid products, containing few illustrative cases, and being totally devoid of the startling and magnificent theories in which microscopists are too apt to indulge. It is divided into four parts, the first being devoted to the consideration of inflammatory products, the second to that of tuberculosis, the third to the typhous process, and the fourth to cancer.

The products of inflammation are separately considered according as they affect:—

1. Serous membranes.
2. Mucous membranes.
3. The external skin.
4. Muscular fibre.
5. Nervous tissue.
6. The organs of respiration.
7. The organs of digestion.
8. The urinary organs.
9. The generative organs.

*Inflammatory products of serous membranes.* The products of inflammation of the pleura are first considered, and are separately discussed according as they are perfectly fluid, coagulated, membranous, or stringy. From his observations on the membranous variety we extract the following notice of the cysts occasionally found free in the pleural cavity.

"The formation of free cysts in fluid exudation of the pleura is a higher form of development. The cyst is inclosed in a white, transparent tissue, similar to the pia mater; it consists of an external layer of thin, transparent, areolar tissue, and internally of a layer of closely crowded cells, whose diameter is about ·007 of a millimeter, and presenting three or four nuclei. Between the layers of areolar tissue, a capillary network may be seen. The pathological formation of these cysts is as interesting as it is obscure. Their contents differ in no respects from the fluid exudation surrounding them. If we regard these perfectly isolated structures as separate and distinct products from their earliest formation, then we must admit that blood-vessels are formed in a cell-membrane, which is in no way connected with the normal parts, and that the isolated structure endowed with an independent vitality generates organic elements. But taking into consideration the analogical production of blood-vessels in other morbid structures, it is very probable that the cyst in an earlier stage of its development took its origin from the investing membrane of the lungs or ribs, and that the blood-vessels have developed themselves from the capillaries on this membrane, and that at a later period separation has been effected." (p. 5.)

Nucleated and elastic fibres are the highest forms of the inflammatory products of the pleura; in the disintegration of these forms into inorganic elements, we observe, in the first place, pigment-cells, then crystals, and finally, larger concretions of crystalline and amorphous constituents.

The fluid exudation in the pericardial sac in cases of inflammation, contains only a few blood-corpuscles, but is the cytoblastema of very numerous and regularly shaped cells, varying in diameter from ·0075 to ·01 of a millimeter having nuclei and nucleoli, and a great number of minute vesicles strewed over their surface. These cells are for the most part aggregated in great numbers; and this circumstance, combined with the uniformity of their structure, explains the rapid development which exudations of this nature undergo.

Plastic exudations in the pericardium assume the most varying forms; they may give rise to membranes, causing the heart to adhere to the free surface of the pericardium; to doubly fusiform bodies (Doppelzapfel), with one extremity attached to the heart and the other to the pericardium; or to a more or less rough investment of the contiguous layers of the pericardium. In colour they vary from a glistening white to a clear red, and they most commonly possess the consistence of coagulated albumen. They consist of a network of coagulated fibres, interspersed with nucleated cells, varying in diameter from ·007 to ·01 of a millimeter. The fibres are characterized by their great transparency and the inequality of their diameters. The cells at many spots are arrested in their development.

The plastic exudations from the serous investment of the interior surface of the heart—the endocardium—may be arranged in two groups. The former embraces those changes which give rise to thickening of the margin of the valves, and finally result in atheromatous and earthy deposits. On instituting a microscopic investigation of this form of exudation, we observe, between the elastic fibres which constitute the normal constituents of the endocardium, a series of undulating cylinders lying parallel to one another, and having a diameter of ·005 of a millimeter, and, between them, fusiform cells, with minute semi-transparent nuclei. The occurrence of these fusiform cells shows the tendency to the production of fibrous structure. The earthy concretions which are formed on the breaking up of the inflammatory products in the endocardium, frequently occur as uncrystallized, amorphous masses, but as frequently they present perfect crystals. These depositions are often formed on the mitral valves, giving rise to thickening of those structures. They likewise occur on the aortic valves, and in this case Günsburg believes that they are produced directly from the earthy salts of the blood. These concretions consist of carbonate of soda, and carbonate and phosphate of lime.

The second group of exudation-structures occurring in the endocardium, is that in which the new elements are not imbedded in, but merely agglutinated to the primary tissue. These are most commonly found on the free edges of the tricuspid valves; they vary from the size of a hemp-seed to that of a pea, are frequently collected in patches, and are tinged red by the blood. They consist of a membrane composed of cells, with a diameter of ·01 of a millimeter, and covered at points with angular epithelial cells.

In his remarks on the products of peritoneal inflammation, he observes, that the serous membrane investing the liver is frequently infiltrated with a solid exudation, and is adherent to the diaphragm by newly formed membranous structures, consisting of very fine elastic fibres lying parallel to each other in irregularly undulating fasciculi, which are inclosed in a structureless membrane of their own. Opaque nuclei may be observed arranged lengthwise on their fibres, and likewise minute vesicles, giving the whole a mottled appearance. Vessels filled with blood-corpuscles may be observed between the fibres.

The peritoneal surface of the spleen is a frequent seat of inflammatory products. Günsburg mentions a case in which the convex surface was covered with a membrane of the thickness of a line, and so puckered and wrinkled as to present a glistening scaly appearance.

The peritoneal coat of the kidneys is rarely the seat of infiltrated exudation, while that of the ovaries is very frequently affected in this manner. Amongst the various changes in the ovary to which plastic exudation gives rise, we may specially notice the formation of cysts which occur either directly on the surface, or are attached to the organ by a pedicle. They are altogether distinct from the cysts, which occasionally are produced in the tissue of the ovary, but they sometimes attain a considerable size, and their walls are formed of a network of nucleated fibres, on which blood-vessels may be detected. The fluid within these cysts contains a small number of cells, having a diameter of ·01 of a millimeter, whose capsules are soluble in acetic acid, and which possess 3—5 distinct nucleoli.

*Inflammatory products of mucous membranes.* The account of the exudation from the nasal mucous membrane is very complete. In the early stage of inflammation it occurs as opaque, whitish, viscid, semi-fluid matter, containing cells of ciliated epithelium, very angular pavement epithelium, stringy mucus, and perfectly spherical nucleated cells, with a diameter varying from ·01 to ·015 of a millimeter, and containing 3 or 4 opaque nucleoli. When the inflammatory process has attained more of a chronic character, the effusion becomes more liquid, although it retains its viscid, stringy character. The secretion then consists of inflammatory cells, varying in diameter according to the degree of their development from ·005 to ·015 of a millimeter. They contain one or two nuclei, presenting several nucleoli. These cells and free nuclei are suspended in great abundance in the structureless viscid mucus.

The formation of nasal polypi is described in the following manner. In chronic inflammation of the Schneiderian mucous membrane, the exudation at certain points undergoes an individual or independent organization. The adherent portion or root of the polypus consists of fusiform cells with elongated nuclei, resembling the ordinary primitive cells of muscular fibre; between these there are deposited nuclei, in some of which a central nucleolus may be detected. It is from these nuclei that the fibrous cells are formed, and they are in structure identical with the nuclei visible in these cells. In making a section of a nasal polypus, we usually find its structural elements arranged in the following order. Externally there is a layer of polygonal cells of pavement-epithelium, forming a protective investment. Below the epithelium is a layer of the round nuclei of the abovementioned fusiform cells, and in most cases below these is a thick layer of nuclei and cells—the organized product, on which depend its shape and firmness. The red vascular structure in the body, and at the extremity of a polypus, consists of a loose network of nucleated fibres, and of capillaries filled with blood and having a diameter not exceeding ·005 of a millimeter.

Günsburg's remarks on the inflammatory products of the mucous membrane of the rectum are illustrated by the following singular case:—

"A girl had severe peritonitis, which, as it gradually yielded, was followed up by an abundant purulent exudation from the mucous membrane of the rectum in the vicinity of the anus. This exudation consisted of inflammatory cells, varying in diameter from ·005 to ·01 of a millimeter, with circular nuclei, and from three to six nucleoli. Some of the cells even attained to a diameter of ·015 millimeters, and contain from ten to eighteen of these nucleoli. The whole mass was neutral, and remained unchanged in concentrated acetic acid, but when the acid was diluted the capsules gradually disappeared. Ether acted in a similar manner. With distilled water heated to 212°, the cells remained unaltered, but with acetic acid at the same degree of heat some of the cell-capsules disappeared, while the nuclei became swollen, and presented an irregular appearance at their edges. Some of the cells were reduced in volume by the boiling acetic acid, and measured only ·005 millimeters." (Vol. i, pp. 23-4.)

In a note to this case Günsburg observes, that on the same day on which the discharge appeared from the rectum, the urine also presented an abundant glistening deposit, and he regards these simultaneous phenomena as indicative of a crisis. The sediment consisted of epithelial cells, whole and broken, in which might be observed many spherical corpuscles, numerous dendritic crystals (urate of soda), and roundish cells, varying in diameter from ·005 to ·007 of a millimeter, and containing three or four minute black nucleoli. In addition to the increased secretion of uric acid—a very frequent occurrence at a crisis—the mucous membrane of the urinary organs presented an inflammatory product capable of plasticity. The simultaneous formation of exudations in the mucous membranes of the urinary apparatus and rectum at the moment of the crisis indicates that the tendency to exudation in the blood was then limited to points at which they (the exudations) could be eliminated by the natural passages without injury to the system. The extinction of the process of exudation in the peritoneum, and its simultaneous appearance at the extremities of the mucous membranes of the intestine and urinary organs, form a natural crisis by antagonism. Facts of this nature are not merely valuable in a pathological view, but are of high import in relation to therapeutics, since they lead us to the knowledge of the time and manner in which we can serviceably apply artificial counter-irritation.

The section terminates with an account of his observations on urethral and vaginal discharges.

*The inflammatory products of the external skin* next claim our attention. Our author considers—first, those which are deposited in the rete mucosum, and which break through the epidermis; secondly, those which are deposited in the intermediate cellular layer (of Henle), and gradually work their way through the rete mucosum and finally to the surface; thirdly, those which are originally deposited in the derma, and at the same time involve the superior layers; and, fourthly, cutaneous ulceration depending on a morbid condition of the blood.

Under the first head he places—

*a.* Inflammatory products after a partial, mechanical destruction of the epithelium and rete mucosum—traumatic inflammation. They consist of inflammatory cells, varying in diameter from ·007 to ·01 of a millimeter, and containing few nucleoli.

*b.* Those in erysipelas bullosum, where there are well-filled inflammatory cells, such as may be observed in the fluid of blisters produced artificially.

*c.* Those in eczema, which is caused by the uplifting of the cuticle at certain points through the deposition of a few inflammatory cells in the rete mucosum. If the inflammatory product is so abundant as to break through the layer of epidermis, it hardens on the surface and forms crusts; it likewise penetrates into the intermediate cellular layer. This is the origin of eczema impetiginosum.

*d.* Itch-vesicles come under this head. Günsburg regards the question respecting the true connexion between scabies and the acarus as one to which we cannot at present give a decided answer.

*e.* Acne, which depends on the deposition of inflammatory cells in the rete mucosum, around the mouths of the sudaminous ducts, giving rise to an hypertrophied deposition of epithelial cells, and thus causing a thickening of the walls of the aforesaid ducts.

Under the second head we have phlegmonous inflammation of the skin, and pemphigus. He describes the latter as forming "in the glands of the hair-sacs, which are partially deposited in this (the intermediate cellular) layer. Instead of the fat-vesicles, or more correctly speaking, from them and from the plasma yielded by the adjacent blood-vessels, there are formed inflammatory cells, which become deposited in the adjacent cellular tissue and rete mucosum. After the removal of the exudation, the hairs fall out, and leave minute depressions in the cuticle after the disease is cured." (Vol. i, p. 29.)

Under the third head there are placed, furunculus, ecthyma, acne indurata, rupia, variola, and varicella; and under the fourth, purulent exudations from scrofulous, syphilitic, and cancerous ulcers. There is nothing on these subjects calling for special notice.

Passing over the sections on the inflammatory products of muscular fibre, and of the nervous centres (the latter having been fully considered by our countryman Dr. J. Hughes Bennett), we proceed to "inflammatory products in the respiratory organs." We extract the following observations on the varieties of glands found in the mucous membrane of the larynx.

"1. Simple grape-shaped glands, filled with cells presently to be described, and devoid of an excretory duct.

"2. Ramifying grape-shaped glands.

"3. Grape-shaped glands with an excretory duct. The cells investing the glands studded with barbed and curved cilia.

"4. Simple flask-shaped glands, formed of several epithelial cells lying over one another, and filled with inflammatory cells. On their apices are one or more ciliated cells.

"5. Double flask-shaped glands, or several simple glands lying on one another, sometimes having an excretory duct in the form of an inverted cone or cup.

"The glands often attain to a length of ·25 and a breadth of ·3 of a millimeter. They are perfectly full of cells, varying in diameter from ·007 to ·01 of a millimeter, with a nucleus and several nucleoli, which entirely prevent us from distinguishing the construction of the glands. After their removal, the tissue is seen to be composed of accurately-fitting cells of pavement epithelium, with nuclei having a diameter of ·005 of a millimeter. Sometimes there are also removed the nuclei of glandular epithelium, analogous in all respects to inflammatory cells, and the glands then exhibit simply a homogeneous membrane strewed with blackish specks.

"The cylindrical epithelium to which the cilia are attached, the pavement epithelium, and those cells which are newly formed in the glands (the enchyma of Purkinje), constitute the greater portion of the expectoration in cases of inflammation of the mucous membrane of the larynx." (Vol. i, pp. 46-7).

These various forms are well illustrated by diagrams.

We now arrive at what may perhaps fairly be deemed the most important subject of the work—*the inflammatory products occurring in the lungs.*

Günsburg observes, that during the first days of pneumonia—the first and second days—the expectoration often contains no blood-corpuscles. The inflammatory cells contain a pale nucleus with a central nucleolus, the nucleus being rendered more apparent by the action of an acid; these cells are filled with minute globules, varying in diameter from ·001 to ·002 of a millimeter. They occur sparingly in almost every expectoration, and are the products of the irritated mucous membrane of the larynx and bronchi, from which source also are derived the epithelial cells, which are constantly present. Pneumonic expectoration submitted to boiling heat becomes converted into a brown caseous mass, and heated with acetic acid yields a brownish red precipitate.

With respect to the changes which occur in the lungs in the progress of inflammation, we find that in incipient pneumonia the pulmonary vesicles are much distended and filled with inflammatory cells, having a diameter of ·005 of a millimeter, and full of granules.

The exudation occurring in the stage of red hepatization, consists of very numerous inflammatory cells, perfectly round, varying in diameter from ·01 to ·015 of a millimeter, and containing from 2 to 5 minute, well-defined nuclei.

The exudations occurring in the stage of gray hepatization are formed of inflammatory cells, varying in diameter from ·005 to ·015 of a millimeter, with a circular nucleus and central corpuscle; also of a small number of granular cells. Between the inflammatory cells there are deposited minute vesicles, fat-globules, or molecules. Acetic acid dissolves the capsules of the inflammatory cells in the pulmonary vesicles, leaving merely the nuclei; while the granular cells remain unchanged. At those parts of the lung in which the pulmonary vesicles are completely destroyed, nothing can be seen between the bronchial fibres but granular and inflammatory cells and the oval, caudate, fusiform cells arising from them, either isolated or arranged in the form of continuous fibres.

The following questions especially suggest themselves in this inquiry.

Which element of the pulmonary tissue is the primary seat of the exudation? And what changes do the other elements subsequently undergo? The following are Günsburg's replies to these questions:

"It is but rarely that the inflammatory cells are distributed in a regular plane between the bronchial fibres; in these cases the pulmonary vesicles appear compressed and to have lost their contents. Most commonly the pulmonary vesicles are the seat of the exudation. They become distended to three or four times their proper size, and inclose great numbers of inflammatory cells, varying in diameter from ·05 to ·08 of a millimeter. In external appearance they are exactly similar to the formative cells of the lung in the embryo, and only differ from them in the circumstance that the nuclei of the latter are insoluble in water and acetic acid, while the inflammatory cells in the lungs undergo the ordinary changes with these reagents. As the disease extends, the walls of the pulmonary vesicles give way, and the inflammatory cells are then only limited by the elastic fibres.

"When the exudation is scanty, the capillaries are seen to be distended with blood; with the augmentation and extension of the exudation they disappear. The exudation sometimes entirely fills the pulmonary vesicles, and is limited to them.

"The pulmonary vesicles and the intercellular walls of the capillaries are the primary seat of exudation. The augmentation of volume of the whole lung is essentially dependent on the distension of the individual pulmonary vesicles, and on their being filled with exudation-cells. I have not observed any thickening of the epithelium of the mucous membrane, even after long standing induration of the exudation. The sphere of development of inflammatory cells is limited, since the excess of exudation is accompanied by a deficiency of blood in the tissue. Moreover, the elastic walls of the pulmonary vesicles do not aid in their further formation. In the stage of purulent infiltration the pulmonary vesicles are seen to be partly filled with granular and inflammatory cells, and partly lying free amongst these two kinds of cells. Whether the exudation is originally poured forth into the intercellular spaces, or whether it does not find its way there till after the destruction of the walls of the pulmonary vesicles, or, in other words, whether there is an essential distinction between vesicular and interstitial pneumonia, are questions to which, with our present results, we can give no satisfactory answers." (Vol. i, pp. 59-60.)

The section terminates with remarks on the microscopic appearances presented by the lungs in gangrene, emphysema, and compression.

*Inflammatory products in the digestive organs* next claim our attention. The following case of typhlitis stercoralis is interesting in several respects:

"The walls of the cæcum were of double their ordinary volume; the peritoneal coat was covered with vessels full of dark blackish blood, especially towards the iliac end. The appendix vermiformis on being opened presented a horseshoe shape; the mucous membrane of its lower portion was of a pale gray colour, thickened and presented numerous minute depressions (grübchen), some of which were isolated, while others were associated into a sort of network. The upper portion was divided by a band into two diverticula, in each of which the mucous coat was converted into a gangrenous capsular membrane, inclosing a hard concretion. The concretion in the lower diverticulum had the form and size of a date-kernel, and was of a bright yellow colour, but soon became brown on exposure to the air.

"The diseased surface was covered by a thin layer of finely granular, yellowish-green matter, in which were detected,—1. A large number of four-sided rhombic prisms, scarcely averaging ·02 of a millimeter in length. 2. Fragments having the form of imperfectly round nucleoli, and in some instances surrounded by a capsule. 3. A quantity of cylinders anastomosing at certain points, and presenting occasional contractions and dilatations. These cylinders or tubes were both filled and surrounded with spherical corpuscles, having a diameter of ·005 of a millimeter and by immeasurably minute molecular granules, which when accumulated presented a blackish-green appearance. On this layer of finely granular matter there was a brownish, very injected membrane. In the fibrous network there were numerous engorged capillaries, inflammatory cells, and crystals. The peritoneal coat, which could not be very clearly made out, was covered with crystalline fragments, and penetrated by numerous injected vessels; inflammatory cells could be observed between its elastic fibres.

"Hence typhlitis stercoralis is characterized by an extremely rapid exudative inflammatory process, occurring simultaneously in all the coats of the affected part, and dependent on mechanical irritation. To this follows gangrene, in consequence of the destruction of the supplying vessels, and hence the disintegration, not only of the normal elementary constituents, but also of the inflammatory products, into molecular particles. At the same time the inorganic elements of these tissues assume the crystalline form." (Vol. i, pp. 69-70.)

The section on *the inflammatory products occurring in the liver* is illustrated by descriptions of the microscopic appearances in three cases of nutmeg, and nine of fatty liver in various stages. From a review of these cases it appears that in all of them there was distension of the capillaries. At the same time fat-globules appeared, at first scanty and afterwards in great numbers, diminishing by their compression the adjacent hepatic cells. Other cells increased in size from their nuclei being apparently converted into fat, in consequence of which the capsules finally dissolved. The amount of fat-vesicles stands in a constant relation with the rarefaction of the hepatic cells, which finally become much compressed and have their interstices filled with matter apparently consisting of nuclei.

In other cases the fatty liver appears to depend, not on the deposition of fat-vesicles externally to the hepatic cells, but in the conversion of the nuclei and the cells themselves into fatty matter; these exert a mechanical influence on the adjacent cells not essentially altered. The fulness of the capillaries, their distension, and the primary swelling of the cells are phenomena which also occur at the commencement of inflammation. From this formation all the material that would be employed in the production of inflammatory cells undergoes a retrograde metamorphosis into fat-globules. The simultaneous conversion of the nuclei of the hepatic cells into fat, their being tinged at certain parts of the liver with bile, and the occurrence of extravasations of bile seem to point out that the primary condition for this formation of fat must be sought in a chemical change in the composition of the blood, and in some abnormality in the secretion of the bile. The further changes leading finally to atrophy must be referred to a combination of mechanical causes acting from without, and a simultaneous conversion of the parts themselves into fat.

The granular, atrophied, fatty liver is described as due to a combination of inflammation and the formation of fat. The granulations consist, for the most part, of hepatic cells of a polygonal outline, varying in diameter from ·01 to ·025 of a millimeter, and, with the exception of containing a few fat-globules, being perfectly empty. Other cells in these granulations contain an enlarged nucleus filled with agglomerated corpuscles; while others are so full of fat-globules as to resemble granular cells; and others again are totally empty. Between these hepatic cells, a few caudate, elongated, fibrous cells may be observed.

The structure occurring between the granulations consists of nucleated fibres running in various directions. In the meshes of the network formed by these fibres, spherical cells, with a diameter of ·005 of a millimeter, and containing several globules, may be observed. These are the remains of hepatic cells. The production of this form of diseased liver is explained by Günsburg in the following manner.

Around the larger vessels of the liver we meet with an inflammatory product succeeding to hyperæmia, and rapidly assuming a higher state of organization. The hepatic cells, inclosed by the fibrous cells and nucleated fibres, are no longer capable of secreting bile, in consequence of the deficient supply of blood they receive, as well as of its altered condition, and they become converted into fat-globules. That inflammation is the primary cause of this affection, and that its products lead to the formation of fat in the liver, seems proved by the facts (deduced from the examination of cases) that all the phenomena of inflammation of the liver are originally present, and that it is not until the symptoms of chronic disease of the liver have existed for a considerable period that dropsy, the invariable attendant on the granular fatty liver, presents itself. Further, the absence of free fat-vesicles and the presence of some uninjured hepatic cells in the large granulations show that the fat in the hepatic cells is formed from the nuclei, and that this abnormal metamorphosis stands in certain relations with, and is consequently dependent on the degree to which the inflammation had extended.

*The inflammatory products occurring in the kidney* are divided by our author into the three following classes.

In the first class he places those in which the product occurs in the genuine form of spherical inflammatory cells, or after a prolonged time becomes formed into granular or fibrous cells. The second class embraces those in which the inflammatory product takes the form of fibrous cells and fibres, causing displacement of the normal tissue, and compressing, or even destroying the urinary canals. This includes several varieties of Bright's kidney, namely, the first, second, third, and sixth forms in Rokitansky's arrangement.*

In the third class the inflammatory product does not assume the cellular form, but the elements of the cell-formation are broken up into masses and destroy the tissue of the organ, giving rise to fatty degeneration of the kidneys. This morbid change was first described by Gluge, but the dis-

* See Vol. XV, pp. 100-102, of this Journal.

covery seems to have been independently made by Günsburg. The following is our author's description of it:

"The kidney is about half as large again as in the healthy state, and presents a greater degree of resistance. On cutting through it we observe that it presents a yellowish-white colour, with reddish streaks, (externally resembling Rokitansky's second form of Bright's disease.) Three days after the examination, the naked eye may detect the escape of fat. On a microscopic examination, innumerable fat-globules having a diameter of ·005 of a millimeter may be at once observed. Between these, perfect granular cells having a diameter of ·02 of a millimeter may be detected. The perfect condition of these granular (or pus-) cells, shows that the formation of fat is dependent on elements which have had a tendency to exudation. The urinary canals appear, for the most part, as transparent empty cylinders.... The development of fat-globules, and the emptying and compression of the urinary canal, are characteristic of fatty degeneration. It differs from the fatty degeneration of other organs, in there being no free effusion of fat on its cut surface." (Vol. i, p. 91.)

The first part of the volume terminates with the *inflammatory products occurring in the generative organs,* under which are considered ovaritis, exudation in the follicles, serous cysts, ovarian dropsy, apoplexy of the follicles, the formation of pigment in them, their cicatrized condition, inflammation of the fallopian tubes, and fibroid of the uterus. The chapter contains several paragraphs that we had marked for insertion, but the length to which our review has already extended, warns us that we must proceed at once to the second division of our volume, which is devoted to the subject of *Tuberculosis,* and occupies the succeeding fifty-two pages.

Passing over the first two chapters of this division, devoted to the tuberculous products of serous membranes, and tuberculosis occurring in the nervous centres, we arrive at the chapter on the tuberculous products of the respiratory organs.

Günsburg commences by observing that, notwithstanding the endemic intermittent fever of the Silesian valleys, tuberculosis of the air-passages, is remarkably frequent, and decimates the population.

Simple tubercular granulations of infiltrated pulmonary tubercle consist, according to our author, of cells having a diameter of ·005—·0065 of a millimeter containing three, five, or even more distinct, minute granules, without, in most cases, the appearance of a nucleus. These cells are unacted on by water and dilute acetic acid, but in concentrated acetic acid the capsule becomes paler and finally dissolves. The blood-vessels in the surrounding pulmonary tissue are much distended, and around them we observe pigment-cells and free granular pigment.

The softening of pulmonary tubercle, the mode of formation and the contents of vomicæ, the expectoration, and the healing process, all meet with full consideration from our author.

The microscopical and chemical characters of the expectoration are recorded in eight cases of phthisis in various stages. At the commencement of tuberculosis few inflammatory or tubercular cells are discharged with the expectoration. That the inflammatory product arises for the most part from the pulmonary vesicles, and the finest ramifications of the bronchial glands, seems obvious from the circumstance that none of the epithelial structures are present; which, as the disease progresses, are thrown off from the bronchi, trachea, and larynx.

The tubercular cells occurring in the expectoration during the early stage of the disease are not so perfect as those occurring in the tubercular granulations themselves.

When the disease extends to the bronchi and larynx, we have, as additional elements of the expectoration, ciliated and pavement epithelium; and when softening commences, molecular fragments of disintegrated tubercular cells are seen intermingled with other intact cells of the same character. At a subsequent stage—when the pulmonary tissue is being rapidly destroyed—we find that the expectoration likewise contains fragments of bronchial fibres, pigment-granules, and minute, generally rhombic, crystals.

In the remaining chapters, devoted to tuberculosis in the digestive and urinary organs, in the muscles and bones, there is nothing requiring especial notice.

The third division of the work treats of the typhous process, a subject we noticed at some length in our review of Rokitansky's 'Pathological Anatomy,' in Vol. XV, pp. 95-8, and to which we shall probably advert in reviewing the first volume of that extraordinary work.

The concluding division of the work may be very briefly dismissed. In the fifty-two pages of which it consists, we have full accounts of the microscopic appearances presented by the various forms of cancer occurring in the liver, stomach, pancreas, spleen, serous membranes, brain, muscle, bone, and external skin. The subject of cancer has been so copiously discussed in the recent numbers of this Journal, that we shall make no extracts from this division; we cannot, however, part with our author, without strongly recommending this, as well as the other portions of his work, to all who devote any attention to the microscopic investigation of morbid products.

---

